# Predictive factors of dropout from inpatient treatment for anorexia nervosa

**DOI:** 10.1186/s12888-016-1010-7

**Published:** 2016-09-30

**Authors:** H. Roux, A. Ali, S. Lambert, L. Radon, C. Huas, F. Curt, S. Berthoz, Nathalie Godart, the EVHAN Group, Nathalie Godart, Sylvie Berthoz, Christophe Lalanne, Jeanne Duclos, Lama Mattar, Hélène Roux, Marie Raphaelle Thiébaud, Sarah Vibert, Tamara Hubert, Annaig Courty, Damien Ringuenet, Jean-Pierre Benoit, Corinne Blanchet, Marie-Rose Moro, Laura Bignami, Clémentine Nordon, Frédéric Rouillon, Solange Cook, Catherine Doyen, Marie-Christine Mouren Siméoni, Priscille Gerardin, Sylvie Lebecq, Marc-Antoine Podlipski, Claire Gayet, Malaika Lasfar, Marc Delorme, Xavier Pommereau, Stéphanie Bioulac, Manuel Bouvard, Jennifer Carrere, Karine Doncieux, Sophie Faucher, Catherine Fayollet, Amélie Prexl, Stéphane Billard, Francois Lang, Virginie Mourier-Soleillant, Régine Greiner, Aurélia Gay, Guy Carrot, Sylvain Lambert, Morgane Rousselet, Ludovic Placé, Jean-Luc Venisse, Marie Bronnec, Bruno Falissard, Christophe Genolini, Christine Hassler, Jean-Marc Tréluyer, Olivier Chacornac, Maryline Delattre, Nellie Moulopo, Christelle Turuban, Christelle Auger

**Affiliations:** 1 Département de Psychiatrie, Institut Mutualiste Montsouris, 42 boulevard Jourdan, 75014 Paris, France; 2 Faculté de Médecine, Université Paris Descartes, Paris, France; 3 Center of Research in Epidemiology and Population Health, INSERM U1018, Paris Sud University, 97 Bd de Port-Royal, F-75679 Paris, France; 4 Université Paris Descartes, Paris, France; 5 Université Paris Sud, Villejuif, France; 6 UVSQ, Villejuif, France; 7 Université Paris-Saclay, Villejuif, France; 8 Service d’Addictologie, CHU Nantes, Paris, France

**Keywords:** Dropout, Anorexia nervosa, Inpatient, Treatment, Predictive factor

## Abstract

**Background:**

Patients with severe Anorexia Nervosa (AN) whose condition is life-threatening or who are not receiving adequate ambulatory care are hospitalized. However, 40 % of these patients leave the hospital prematurely, without reaching the target weight set in the treatment plan, and this can compromise outcome. This study set out to explore factors predictive of dropout from hospital treatment among patients with AN, in the hope of identifying relevant therapeutic targets.

**Methods:**

From 2009 to 2011, 180 women hospitalized for AN (DSM-IV diagnosis) in 10 centres across France were divided into two groups: those under 18 years (when the decision to discharge belongs to the parents) and those aged 18 years and over (when the patient can legally decide to leave the hospital). Both groups underwent clinical assessment using the Morgan & Russell Global Outcome State questionnaire and the Eating Disorders Examination Questionnaire (EDE-Q) for assessment of eating disorder symptoms and outcome. Psychological aspects were assessed via the evaluation of anxiety and depression using the Hospital Anxiety and Depression Scale (HADS). Socio-demographic data were also collected. A number of factors identified in previous research as predictive of dropout from hospital treatment were tested using stepwise descending Cox regressions.

**Results:**

We found that factors predictive of dropout varied according to age groups (being under 18 as opposed to 18 and over). For participants under 18, predictive factors were living in a single-parent family, severe intake restriction as measured on the “dietary restriction” subscale of the Morgan & Russell scale, and a low patient-reported score on the EDE-Q “restraint concerns” subscale. For those over 18, dropout was predicted from a low depression score on the HADS, low level of concern about weight on the EDE-Q subscale, and lower educational status.

**Conclusion:**

To prevent dropout from hospitalization for AN, the appropriate therapeutic measures vary according to whether patients are under or over 18 years of age. Besides the therapeutic adjustments required in view of the factors identified, the high dropout rate raises the issue of resorting more frequently to compulsory care measures among adults.

## Background

Anorexia Nervosa (AN) is a serious psychiatric pathology associated with a high rate of mortality [26]. Emergency hospitalization is required in the most severe cases, when ambulatory treatment has failed, or when the condition becomes chronic [1, 14, 21].

Hospitalization thus selects patients with a poor prognosis [16], and a large number of them do not comply with set care objectives, and drop out prematurely from inpatient treatment [36]. Leaving the hospital before care is complete (i.e., before the target weight is reached) predicts poor outcome and increases the risk of relapse within the year [2, 5, 28]. Patients who have dropped out from inpatient care also display more eating disorder (ED) symptoms at follow-up [2], and a more chronic and serious course of illness. Conversely, it has been shown that compliance facilitates recovery and successful treatment [32].

To our knowledge, only ten studies in the literature [16–18, 22, 24, 27, 30, 33, 38, 40] have focused exclusively on dropout in populations of patients hospitalized for AN.

Results from these studies (see Table 1) are not easy to compare because the definition of dropout from inpatient care varies, and the study populations differ, in particular with regard to age. Eight of the studies mainly involve adult samples and only one includes solely adolescents and very young adults (mean age 16.6, sd = 1.9) [17]. Dropout rates can reach 56.2 % of adults hospitalized for AN and 24 % of adolescents, suggesting that dropout is less frequent among children and adolescents than among adults. It can be noted that in France, the decision to hospitalize legally belongs to the parents when the patient is under age 18, while among adults (≥18), it is the patient who decides. All existing studies are single-centre studies except for one [30], and their results have never been replicated in larger multi-centre samples. Based on previous studies (see Table 1), the aims of the current study were, in a multi-centre study, (1) to compare the risk of dropout in adolescents and in adults, and (2) to explore the predictors of premature dropout from inpatient treatment for AN, taking into account all previously identified factors, so as to determine clinical signs that could alert the clinician to the risk of dropout, and provide relevant targets for treatment.

**Table 1:** Sum-up of the litterature about drop in treatment for anorexia nervosa

	Vandereycken and Pierloot (1983) [33])	Kahn and Pike (2001) [18])	Woodside et al. (2004) [38]	Surgenor et al (2004) [30]	Zeeck et al. (2005) [40]	Godart et al. (2005) [11]	Carter et al. (2006) [6]	Huas et al. (2011) [16]	Hubert et al. (2013) [17]	Sly et al. (2014) [27]	Pham-Scottez et al. (2014) [24]
Multi-centre study	No	No	No	Yes	No	No	No	No	No	Yes	No
Number of patients included	133 women	81 women	166 men and women	213 men and women	133 men and women	268 women	77 women	601 women	304 women hospitalisations	130 women and 5 men	64 women
Age of patients m(Sd)	20.5 (4.8)	26.3 (7.4)	27.1 (9)	21.4 (6.6)	24.3 (6.8)	16.7 (2)	25.5 (7.8)	20.5 (4.8)	16.6 (1.9)	28.8 (10.1)	24.9 (5.9)
Definition of dropout	Leaving hospital before end of treatment	Leaving hospital before reaching 90 % ideal BMI	Premature departure at BMI <20, discharge decided by healthcare team in absence of progress or violation of rules	Leaving hospital against medical advice or abandonment of treatment	Decision by patient or healthcare team to abandon treatment prematurely	Weight contract not met or loss of weight	Patient can leave the programme as desired in case of lack of progress or failure to gain weight	Leaving hospital before planned discharge in therapeutic contract. The patient and/or the healthcare team can decide on termination of contract	Weight target fixed in contract not met	Patient who initiated discharge themselves	Any discharge before normal treatment program termination
Predictive factors			Greater maturity fears (EDI) Lesser restraint concern (EDE)	AN-P Lower BMI at admission	Larger number of symptoms at admission (SCL-90R) Absence of diagnosis of depression (DSM-IV)	Higher BMI at admission Lower BMI at discharge Later age at onset, Longer duration of hospitalisation		-Having one or more child -Lower Educational status, - Higher SCL-90 paranoid ideation, - Higher Morgan-Russell food intake subscale - Minimum BMI - Desired BMI - Diuretic use - Laxative use - Previous hospitalization for ED	Living with a single parent Previous hospitalisation for ED lower BMI at admission patient over 18	Having a lack of motivation and alliance	Having a personality disorders in comorbidity with AN (SIDP-IV)

*M* mean, *BMI* Body mass index, *ED* Eating disorders, *EDI* Eating disorders inventory, *AN-P* Anorexia Purging Type, *EDE* Eating disorders evaluation, *SCL-90R* Symptom Checklist-90-R, *Sd* Standard deviation

## Method

### Patient population (see Fig. 1)

**Fig. 1:**
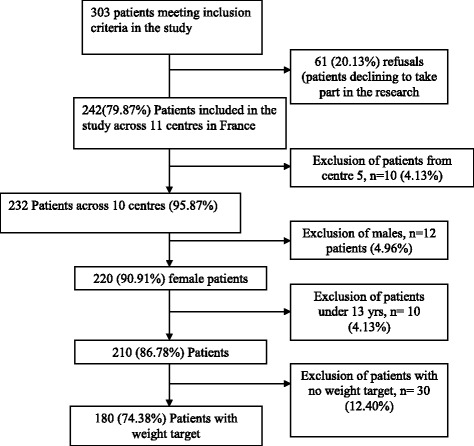
Flow-chart for the EVALHOSPITAM study

#### Subjects and settings

This study was part of a larger study known as EVHAN (evaluation of hospitalization for AN, EVALHOSPITAM in French, Trial registration number: 2007-A01110-53). A total of 242 patients with AN were recruited from inpatient treatment facilities for AN in 11 centres in France between 2009 and 2011 (see list in Annex 1).

Inclusion criteria for the current study were as follows: being hospitalized for AN, admission Body Mass Index (BMI) <15 and/or sudden and rapid weight loss, agreement to participate in the study, and being affiliated to the French Social Security health coverage system. Exclusion criteria were refusal to participate, insufficient command of the French language, existence of a potentially confounding pathology (e.g., diabetes, Crohn’s disease or other metabolic disorders), male gender, being under the age of 13, incomplete psychopathological assessment, and patients hospitalized without any weight target being set. Thus, 180 female patients were included in this study.

Four patients did not have a BMI < 17.5 at admission, however two of them had fallen from a BMI above the 97th percentile to a BMI in the 10th percentile relative to their age in the year prior to hospitalization, and two others had had a BMI < 17.5 in the previous three months but had been admitted into a medicine unit, with resulting weight gain, just before their transfer to the psychiatry unit and inclusion into the study.

A global assessment investigating different aspects of the patients’ psychic and somatic status was performed in the hospital during the first week of admission to inpatient treatment and before discharge. Admission and discharge weights were collected.

#### Inpatient treatment programs

During the study period, patients were hospitalized on a voluntary basis for adults and with parental agreement for those under 18 (i.e., not legally adults*)*. All were hospitalized for a life-threatening physical and/or mental state as recommended in international guidelines [1, 14, 21] (low BMI and/or rapid weight loss and/or compromised vital functions, severe depression, high suicide risk, chronic under-nutrition with low weight, and failure of outpatient care). Failure of outpatient treatment is defined as a significant deterioration or the absence of significant improvement in terms of weight gain, ED symptoms and/or severity of the psychological state. Briefly, the inpatient programs were all multidisciplinary and included somatic, nutritional and psychological treatment goals, in compliance with the French guidelines for ED [14]. All clinical teams involved had collaborated in drafting the French guideline recommendations [14]. The inpatient programs differed slightly according to the centres and the age of the patients. A few of these programs are described in published papers [9–11, 39]. Patients included in the current study had a target discharge weight that was determined at the beginning of treatment. Patient-initiated discharge was defined as any dropout initiated by the patient, and/or her parents for those under 18. Staff discharge refers to instances where the staff decided to discharge a patient who had not yet reached her target weight.

#### Procedure

##### Terminology: definition of dropout

One of the factors that need to be considered concerning dropout is the weight at discharge, since it can highlight resistance or ambivalence on the part of the patient with regard to recovery. Dropout was defined as a decision of discharge before the end of treatment, the definition of which (target weight) was determined at the beginning of the inpatient treatment with the team and the patient/parents in a bilateral agreement. Thus, we operationalized the definition of dropout as “not reaching the target discharge weight” at time of discharge, regardless of whether the patient, the parents or the staff terminated the treatment (staff discharge usually occurred because of a lack of progress over a long period of hospitalisation, i.e., some months of stagnation) [16].

Dropouts were classified either as “early” (before the half-way mark in weight gain between admission and discharge, i.e., with very low weight and consequently high somatic risk) or “late” (after this half-way mark with a better nutritional status), according to the period in which they occurred in the process of weight gain [11].

##### Assessments

Assessments were performed using an electronical notebook called “*Cahier d’observations électronique”* CleanWeb, e-CRF; Telemedecine technologies S.A). The current diagnosis of AN was based on the DSM-IV criteria, the Eating Disorder Examination (EDE-Q, see below for description) [23], and the Composite International Diagnostic Interview (CIDI) 3.0, which is a structured diagnostic interview, along with the following BMI criteria: BMI <10th percentile up to 17 years of age, and BMI <17.5 for 17 years of age and above [37].

Besides information concerning mental state and nutritional status, the evaluation collected socio-demographic data, present weight, minimum and maximum weight with corresponding ages and statures, the last educational level reached, and a global clinical evaluation. The instruments used are described below.

The global clinical assessment used the Morgan & Russell Global Outcome Assessment scale [20] which assesses the clinical state over the previous six months by way of five clinician-rated subscales exploring diet, menstruation, mental state, psychosexual functioning and socioeconomic status. Scores range from 0 (the worst) to 12 (the best).

The Eating Disorder Examination Questionnaire (version EDE-Q-5.2) is a self-report version of the EDE. It is a 28-item self-report questionnaire that focuses on patient report of symptom occurrence over the past 28 days and includes four subscales: “restraint concerns”, “eating concerns”, “weight concerns”, and “shape concerns” [23]. The higher the score, the greater the difficulties reported by the patient.

The Hospital Anxiety and Depression Scale (HADS) is a self-report scale comprising 14 items which assess the most frequent anxiety and depression symptoms. It has become widely established as a convenient self-rated instrument for anxiety and depression in patients with both somatic and mental problems, and with equally good sensitivity and specificity as other commonly-used self-rated screening instruments [4, 15, 41].

### Statistical analyses

The analyses were performed using SPSS 20.0.

Since, in the course of analyses, being over 18 as opposed to under 18 proved to be an important predictor of dropout, the initial sample was split into two subsamples, under-18 and 18+. To describe the sample, frequencies were used for qualitative variables, and means and standard deviations (SD) for quantitative variables. Group differences between the two age groups were assessed with Student *t* test and Chi-2.

The association between premature termination of treatment and potential predictive factors suggested from the literature was investigated in two stages.

First, we tested the link between dropout and all potential predictive and adjustment factors (including centres) using univariate analysis with Kaplan-Meier test to compare the probability to dropout. The log-rank test was used to compare the survival probabilities to dropout of different groups. All the variables described as significant predictors in the literature were tested into the univariate model as independent variables [16, 17, 36]. These included: age at admission as under-18 or 18+, AN subtype, BMI at admission, minimum BMI, amplitude of BMI target (BMI target set by the team for discharge minus BMI at admission), AN illness duration (under or over 4 years, which is considered as the chronicity threshold, Huas et al. [16]), score on the “weight concerns” subscale of the EDE-Q, score on the “restraint concerns” subscale of the EDE-Q, score on the “depression” subscale of the HADS, score on the “dietary restriction” subscale of the Morgan & Russell scale, number of previous hospitalizations for AN. For strongly correlated variables, only that with the “greatest clinical relevance” was retained to avoid collinearity (correlation test was used). As minimum BMI and admission BMI showed a strong positive correlation one to the other (rho = 0.764, *p* < 0.001), admission BMI alone was retained.

Second, three multivariate analyses were done by adjusting Cox regressions with robust estimation on the overall sample and then the two subsamples, under-18 and 18+. To calculate the relative risk of drop-out, hazard ratios (HRs) and 95 % confidence intervals (95 % Cis) were obtained by Cox proportional hazard models. Cox regression analyses were performed in order to explore multivariate relations between dropout and associated variables.’forward sepwise’ (significant model variables with the highest significant score) method was used for multivariate analyses. This analysis considers the follow-up for each patient and assumes that the effects of the predictor variables upon survival are constant over time.

We did not propose an adjustment to the alpha-level (multiple testing) as recommended by Bender and Lange [3] because the study was exploratory and searching for effects, and its design was not suited for testing causal hypotheses. All statistical tests were two-tailed, the level of significance was α = 0.05.

## Results

### Description of the overall sample (see Table 2)

**Table 2:** Description of the overall sample and comparisons between under-18 s and 18+

	Total	(≥18 years)	(<18 years)	Comparisons 18+ with under-18 s
	*N* = 180	*N* = 97	*N* = 83	
	*N* (%)	*N* (%)	*N* (%)	Student’s test or chi-square (*p* value)
Dropout	*n* = 58 (32.22 %)	*n* = 41 (42.26 %)	*n* = 17 (20.48 %)	9.72 (0.004)
AN Type				
Anorexia nervosa Restrictive Type	*N* = 88 (48.89 %)	*n* = 48 (49.49 %)	*n* = 40 (48.19 %)	0.06 (0.81)
Anorexia nervosa Bingeing Purging Type	*N* = 92 (51.11 %)	*n* = 49 (50.51 %)	*n =* 43 (51,81 %)	
	m (sd)	m (sd)	m (sd)	
Age at admission	20.67 (6.77)	24.83 (6.78)	15.82 (1.39)	12.78 (<0.001)
BMI at admission	14.16 (1.42)	14.04 (1.44)	14.30 (1.44)	1. 23 (0.22)
Minimum BMI	13.02 (1.59)	12.62 (1.58)	13.51 (1.48)	3.89 (<0.01)
BMI at discharge	17.19 (2.13)	16.57 (2.27)	17.91 (1.68)	4.51 (<0.001)
BMI target fixed by healthcare team	17.83 (1.76)	17.52 (2.02)	18.15 (1.39)	(2.35) 0.02
Duration evolution AN (months)	49.95 (54.25)	73.40 (63.47)	22.78 (20.55)	6.91 (<0.001)
Number of previous hospitalisations for AN	1.18 (0.82)	1.23 (0.80)	1.12 (0.85)	0.89 (0.375)
Duration of hospitalisation (days)	129.76 (102.49)	123.06 (103.06)	137.6 (101.83)	0.95 (0.344)
Score for “weight concern” EDE-Q subscale	3.69 (1.45)	3. 89 (1.35)	3.45 (1.54)	2.04 (0.043)
Score for “restraint” l’EDE-Q subscale	3.79 (1.81)	4.06 (1.82)	3.47 (1.75)	2.16 (0.032)
Score for “depression” on HADS	9.28 (4.43)	10.11(4.49)	8.31 (4.17)	2.74 (0.007)
Score for “dietary restriction”M&R subscale	1.63 (1.78)	1.41 (1.59)	1.92 (1.96)	1.89 (0.059)

*EDE-Q* Eating Disorder Examination Questionnaire, *HADS* Hospital Anxiety and Depression Scale, *M&R* Global Outcome Assessment Scale, Morgan and Russell

The patients recruited were aged 13–52 years, 46 % were under 18 and 54 % were 18 or over. Half of the patients presented a restricting form of AN, and the others the bingeing-purging subtype. Their clinical state was very severe at admission, with a BMI of 14.16 ± 1.42 kg/m2 for a mean age of 20.67 years (6.77) (that is to say well below the 3rd percentile for the mean age) [25]. Minimum BMI and admission BMI were strongly correlated (rho = 0.674, *p* < 0.001). For 35 % of the patients, the AN illness duration was greater than four years. The target BMI set by the various clinical teams stood at an average of 17.82 (1.76) for an actual BMI at discharge of 17.19 (2.3). The average duration of hospitalization was greater than four months.

### Dropout

Dropout occurred for 32.2 % of the overall sample, amounting to 58 patients. Dropout concerned 20.5 % (17/83) of the under-18 s and 42.3 % (41/97) of the 18+ group. Of these 58 early discharges, 15.5 % (9/58) were on the initiative of the health care team (11.8 % in the under-18 group and 17.1 % in the 18+ group) and 84.5 % were on the initiative of the patient and/or her parents (88.2 % of the under-18 s and 82.9 % of those aged 18+). Among cases of dropout overall, 60 % (35/58) occurred “early” (before reaching the half-way mark towards the target BMI) (53 % for the under-18 group and 62.5 % for the 18+ group) as compared to 40 % that occurred “late” (47 % of the under-18 s and 37.5 % of the 18+).

### Univariate analysis (see Table 3 for details)

**Table 3:** Results of univariate tests for hazard ratio survival models [C1905] and p-values for the sample overall, for under-18 s and for the 18+ group

	Hazard Ratio [CI95]; p		
Variables	Overall sample	18+ group	Under-18 s
Age >18 years	2.26[1.28;3.49]; 0.004		
Age		1.01[0.97;1.06]; 0.547	0.94[0.66;1.32]; 0.712
AN- R (versus AN-B)	1.04[0.62;1.7]; 0.983	1.09[0.59;2.02]; 0.775	0.95[0.36;2.54]; 0.924
Admission BMI	0.87[0.73;1.04]; 0.137	0.88[0.72;1.09]; 0.243	0.91[0.65;1.28]; 0.599
HADS depression score	1.04[0.98;1.11]; 0.17	1.05[0.98;1.11]; 0.141	0.955[0.847;1.077]; 0.452
Dietary subscale M&R	0.97[0.83;1.13]; 0.71	1.03[0.84;1.26]; 0.81	0.953[0.736;1.234]; 0.715
BMI target amplitude	0.81[0.68;0.96]; 0.01	0.79[0.66;0.95]; 0.01	0,958[0.643;1.43]; 0.835
EDE-Q “restraint” subscale	0.89[0.78;1.03]; 0.12	0.89[0.76;1.06]; 0.185	0.805[0.612;1.058]; 0.114
EDE-Q “weight concerns” subscale	0.93 [0.78;1.12]; 0.43	0.86[0.68;1.09]; 0.206	0.942[0.687;1.291]; 0.709
Previous hospitalisation for AN	1.27[0.90;1.79]; 0.38	1.59[1.01;2.47]; 0.1	0.857[0.485;1.515]; 0.596
Duration AN < 4 years	1.94[1.15;3.27]; 0.01	1.43[0.75;2.72]; 0.269	1.559[0.478;5.087]; 0.461
Age at onset of disturbances	1.01[0.95;1.07]; 0.85	0.95[0.89;1.02]; 0.174	0.983[0.79;1.222]0.876
Having at least one child (vs none)		1.66[0.59;4.69]; 0.332	
Educational level: less than *Baccalauréat (*vs *Baccalauréat* or more)		0.64[0.33;1.25]; 0.189	
Living with a single parent (vs living with both)			2.42[0.91;6.45]; 0.069
Centre	1.00[0.92;1.09]; 0.966	0.98[0.88;1.08]; 0.028	0.86[0.69;1.07]; 0.187

*M&R* Global Outcome Assessment Scale, Morgan & Russell

The link between dropout and the potential predictors identified in the literature (see Table 1) was explored using univariate analysis and taking into account the adjustment factors, first for the sample as a whole, and then for the under-18 and the 18+ groups separately.

#### Overall sample

Time to dropout differed significantly between the two age groups (*p* = 0.0004). The probability of dropping out of treatment prematurely was significantly greater among patients in the 18+ group than those in the under-18 group.

The smaller the difference between admission BMI and target BMI set by the health care team (“target BMI amplitude”), the greater the risk of premature dropout (*p* = 0.013) (see Table 3).

#### The 18+ group (see Table 3)

Among patients in the 18+ group (*n* = 97), only the target BMI amplitude and the health care centre were significantly linked to time to dropout (*p* = 0.01 and *p* = 0.028, respectively). No significant results were observed for the other variables studied.

#### The under-18 group (see Table 3)

The time to dropout function for participants under-18 living with a single parent was below that for those under-18 who were living with both parents. Living with a single parent (separated, widowed or divorced) showed a trend towards a link with the time to dropout (*p* = 0.069). No other significant relationships were observed for the other variables studied.

### Multivariate analyses

#### Overall sample (see Table 4)

**Table 4:** Cox model on the whole sample: results and adjusted relative risks

Factors	β	*P*-value	Adjusted relative risk [CI95]
Age group (reference under-18 s)	0.844	0.011	2.326 [1.21; 4.47]
BMI target amplitude	−0.227	0.009	0.797 [0.672; 0.946]
Score on EDE-Q “restraint concerns”^a^	−0.160	0.045	0.852 [0.729; 0.996]
Duration of AN (reference under 4 years)	0.524	0.085	1.688 [0.929;3.067]

^a^Highscore: highly restrictive behaviour

When all the predictive factors identified in the literature were taken into account (see Table 1), the likelihood of dropout was 2.3 times greater among patients in the 18+ group than among those in the under-18 group. It was also found that the narrower the target BMI amplitude and the lower the score on the EDE-Q “restraint concerns” subscale, the greater was the likelihood of premature dropout. The instantaneous risk of dropout showed a tendency towards significance among patients for whom illness duration was greater than 4 years, as compared to those for whom duration was under 4 years.

#### The 18+ group (see Table 5)

**Table 5:** Cox model for the 18+ group – results and adjusted relative risks

Factors	β	*P*-value	Adjusted relative risk [CI95]
Age	0.044	0.069	1.05 [0.99; 1.09]
Score on HADS depression subscale	−0.118	0.005	1.13 [1.04; 1.22]
Score on EDE-Q “weight concerns subscale^a^	−0.374	0.004	0.69 [0.53; 0.89]
Educational level (reference less than the *Baccalauréat*)	−0.91	0.033	0.40 [0.17; 0.93]

^a^High score: patient exhibiting great concern about weight

When the various predictive factors identified in the literature were taken into account, the instantaneous risk of dropout for a patient aged 18 or over was significantly greater when there was a low score on the HADS depression subscale, and a low score on the EDE-Q “weight concerns” subscale. The risk of dropout in the 18+ group was also 2.5 times greater for patients with an educational level lower than a high school diploma than for those with a high school diploma or greater educational level. There was a tendency for the older patients in this group to have a greater instantaneous risk of dropout.

#### The under-18 group (see Table 6)

**Table 6:** Cox model for the under-18 group – results and adjusted relative risks

Factors	β	*P*-value	Adjusted relative risk[CI95]
Score on the M&R “dietary restriction” subscale^a^	−0.484	**0.025**	0.62 [0.40;0.94]
Score on the EDE-Q “restraint concerns”subscale^b^	−0.581	**0.009**	0.56 [0.36;0.87]
Living with a single parent	1.415	**0.021**	4.12 [1.24;13.67]

^a^Low score : very restrictive behaviours

^b^High score : very restrictive behaviours

When all potential predictors identified in the literature were considered, the instantaneous risk of dropout was 4.1 times greater for an under-18 patient living with a single parent than for an under-18 patient living with both parents. A low score on the Morgan and Russell “dietary restriction” subscale and a low score on the EDE-Q “restraint concerns” subscale significantly increased the risk of dropout.

## Discussion

This study aimed to identify factors that are predictive of dropout from hospitalization among women treated for anorexia nervosa (AN). Identifying relevant socio-demographic characteristics and clinical signs that could enable care teams to detect the risk of premature dropout from inpatient treatment could provide preventive therapeutic strategies in order to avoid the consequences of dropout, that are damaging for the future of patients with severe forms of AN.

In the present sample, the overall proportion of patients who dropped out early from inpatient care was 32.2 %. The proportion was significantly smaller among patients in the under-18 group than among those in the 18+ group. This confirms our hypothesis based on previous findings in the literature. The dropout rate of 20.5 % found among the under-18 s is close to that previously reported by the only team to have published data on this topic (24 %) [11, 17]. For patients aged 18 and over, the dropout rate of 42.3 % found in the current study is similar to the rate (between 42 % and 46 %) reported in France by Huas and collaborators [16].

### Overall sample

Among the predictive factors found in the study sample as a whole, the first factor was the age group (under −18 vs. 18+). Being 18 or over at admission significantly increased the risk of dropout from inpatient treatment.

The difference in dropout rate between the under-18 and the 18+ groups can probably be explained by the fact that, across age groups, consent for treatment does not derive from the same sources –for patients under 18, consent must be signed by the parents, while for those aged 18 or older, it is the patient who provides agreement. The persons with AN frequently refuse care because they do not recognize their illness. Adult patients are generally hospitalized in departments specialized in the treatment of AN under their own consent, except in instances of emergency that justify compulsory hospitalization. For adult patients requiring hospitalization but reluctant to agree, this raises the question of whether compulsory hospitalization should be more frequently resorted to in France, when it is justifiable and may indeed be necessary (for instance in the case of life-threatening illness) [31]. Outside of immediate vital risk situations where this indication cannot be disputed, this raises the question of what we know about the long-term effectiveness of this procedure [7]. No patient in the current sample had undergone compulsory hospitalization.

In contrast, individuals under 18 are hospitalized under the responsibility of their parents, and cannot withdraw from care without parental consent. This probably explains why dropout was less frequent in this age group. Nevertheless, the question remains of compulsory hospitalization for the 20 % of under-18 s who dropped out even so. Compulsory hospitalization of adolescents with AN in France is only resorted to in case of imminent danger, and to our knowledge there has been no study published on this topic in the international literature.

The second factor identified, independent of age groups, was a small target BMI amplitude (BMI to be gained before discharge) fixed by the health care team at admission. The target weight chosen partly varies according to the patient’s wishes (and parental opinion for under-18 s) as has been shown in one of the study centres [11]. The more motivated a patient is for hospitalization and treatment, the more readily she accepts weight gain, and the greater the target BMI amplitude. In situations where the therapeutic alliance is problematic, the medical team bargains on the targets to be met, in an attempt to provide at least some treatment for these patients, which is better than none at all given their acute state. Care is then relayed to ambulatory structures or day hospital. Nevertheless, this means that the harder the bargaining and the lower the target set -reflecting resistance to care (from patient and/or parents), the more likely the patient is to drop out prematurely from inpatient treatment.

The third factor identified in the current study showed that the lower the score on the EDE-Q “restraint concerns” subscale (reflecting low patient-reported level of dietary restriction), the greater the probability of premature dropout from inpatient treatment. This is in contrast with a finding from Woodside et al. [38], contrast probably linked to different methods of evaluation: Woodside et al. used the EDE, which is an interview, while we used a self-administered questionnaire. In fact, in our sample of severely under-nourished patients, all had a very low admission BMI resulting from considerable dietary restriction. In this situation, the more reluctant a patient is to recognize her dietary restriction, the more she is at risk. The refusal to recognize the illness indicates a state of denial, and the less willing the patient is to agree to treatment, the more likely she is to drop out prematurely from inpatient treatment.

It should also be noted that no centre effect was observed.

### Adult patients aged 18 years and over

The present study identified three factors in the adult group. The first factor was that a low educational level (no high school diploma) increased the risk of premature dropout from inpatient treatment, confirming the single-centre study conducted by Huas et al. [16].

The second factor identified indicated that a low score on the HADS depression subscale was linked to an increased likelihood of dropping out prematurely from inpatient care. A low score on this scale indicates that there are few symptoms of depression. This result is similar to that reported in the literature [40] – not being depressed is associated with a higher risk of dropping out from treatment. It would therefore appear that depressive symptoms tend to favour acceptance of care.

The third factor indicated that a low score on the “weight concerns” subscale of the EDE-Q (which reflects the absence of perception of difficulties by the patient), also increased the risk of premature dropout from inpatient care. Here again, we believe that the patient’s state of denial explains this result, in line with a finding from an earlier study [8]. In the current sample, we found that patients with low levels of body weight concerns appeared to be in a more severe condition at admission, had a more frequent history of previous hospitalization for AN, a lower BMI objective at admission, and seemed more difficult to manage during hospitalization, with a higher rate of dropout from treatment. This greater risk of treatment failure is consistent with the study by Greenfeld et al. [13]. Given the inconsistency between the low level of symptoms as assessed by self-report measures and the obvious severity of these patients, we wonder whether they are denying their symptoms or whether they lack insight, defined as the ability to acknowledge having an illness or symptoms as well as an awareness of the need for treatment and of the risk of recurrence. Wade [35] contended that AN is particularly associated with denial, which is a response consistent with the egosyntonic nature of the disorder [34]: the more severe the AN, the more severe the denial, and the more likely the patient is to drop out from treatment.

These results nevertheless need to be replicated in future studies.

### Patients under 18 years

Dropout in the under-18 group was linked to three factors at admission which were independent from each other: living with a single parent, scoring low on the “restraint concerns” subscale of the EDE-Q (which in this instrument indicates little awareness by the patient of her dietary restriction) and being given a low score on the Morgan & Russell “dietary restriction” subscale (which indicates serious dietary problems as evaluated by the clinician for the previous 6 months).

Almost one third of the participants under 18 were living with a single parent (divorced, widowed or separated). These patients had a four times greater risk of dropping out prematurely from inpatient treatment. This confirms the result obtained by Hubert et al. [17]. For a single or widowed parent, opposing a child’s demand to terminate treatment could be more difficult, because the parent has to cope with the situation and hold out on his or her own. When parents are divorced, misunderstandings between the former partners could also create problems. An adolescent with AN could be more likely to turn to the parent that is against hospitalization so as to get permission to leave the hospital early. This probably explains why this particular family situation significantly increases the likelihood of premature dropout from inpatient treatment. This is a situation that needs to be recognized at the start of care so as to explain the issues involved, to form a good therapeutic alliance with the parent or parents, to guide and support them, and to back them up in managing conflicts with their child. This would enable them to cope better with the situation where the child wishes to drop out from treatment. This individual support should be bolstered by parent support groups, in which the parents can realize that hospitalization is for the patient’s good, and that resisting dropout is in the patient’s best interest.

We also demonstrated that a low score on the EDE-Q “restraint concerns” subscale, reflecting the absence of awareness of the difficulties by the patient, was a risk factor for premature dropout from inpatient treatment. All the hospitalized patients had a very low BMI at admission indicating severe dietary restriction in the months preceding hospitalization, as seen from the Morgan and Russell score for “dietary restriction”: indeed, when an AN patient reports on the EDE-Q “restraint concerns” subscale that he or she is not restricting diet, this means that the patient has no perception of his/her difficulties, or is in denial of the disorder. Denial probably also contributes to more frequent refusal of treatment or premature dropout – the more unaware the patient is of being ill, the less likely he/she is to accept treatment, and the more readily he/she will drop out of treatment.

One strength of this study is the large number of participants. Limitations are as follows. The number of patients overall and in each group is nevertheless moderate given the number of predictive factors tested. Impulsiveness and personality disorders among the patients were not assessed, while these factors could be linked to the risk of premature dropout from treatment. Given the numbers of patients in each group, we were not able to differentiate dropout linked to the health care team decision from dropout linked to the patient’s decision. Finally, no comprehensive psychopathological evaluation was performed.

## Conclusion

In conclusion, dropout is more frequent among adult subjects than among adolescents. Among the 18+ patients, dropout is so frequent that it raises the issue of the need to apply compulsory hospitalization where useful and necessary (i.e., in case of life-threatening illness [14, 21, 31]), while at present this procedure is rarely resorted to in France. Outside of situations where the eating disorder poses an immediate risk to the individual, we cannot yet determine whether the use of compulsory treatment would positively alter the long-term effectiveness of inpatient treatment. Further, the larger question of the role of inpatient treatment in the continuum of care for AN remains to be addressed. There is a growing body of literature that challenges the cost-effectiveness of long hospitalizations for both adults and adolescents suffering from AN [12, 19, 29]. Chronicity, hopelessness and dismay at the ineffectiveness of treatment are probably at the root of early termination for older patients.

Most importantly, there are certain easily detectable clinical signs and sociodemographic indicators at admission that can serve as warning signals for potential dropout from treatment. Among adult patients, these are a poor awareness of the eating disorder (few weight concerns), a refusal to put on much weight during hospitalization (low target BMI), a low educational level, and the absence of self-reported depressive symptoms. For adolescents, the warning signs are inadequate awareness of the eating disorder alongside patently obvious restrictive symptoms, and living with a single parent (widowed, divorced or separated). These elements that favour dropout suggest the value of developing psycho-educative group sessions centred on symptoms, their recognition, their consequences, and the need for care, with a focus on motivation for change. For adults, particular attention is needed for the less educated patients. For adolescents, greater back-up needs to be provided for single parents in the form of support groups and individual care programs as appropriate.
